# The Influence of Interfering Substances on the Antimicrobial Activity of Selected Quaternary Ammonium Compounds

**DOI:** 10.1155/2013/237581

**Published:** 2013-09-14

**Authors:** Paula A. Araújo, Madalena Lemos, Filipe Mergulhão, Luís Melo, Manuel Simões

**Affiliations:** LEPAE, Department of Chemical Engineering, Faculty of Engineering, University of Porto, Rua Dr. Roberto Frias, 4200-465 Porto, Portugal

## Abstract

Standard cleaning processes may not remove all the soiling typically found in food industry, such as carbohydrates, fats, or proteins. Contaminants have a high impact in disinfection as their presence may reduce the activity of disinfectants. The influence of alginic acid, bovine serum albumin, yeast extract, and humic acids was assessed on the antimicrobial activities of benzalkonium chloride and cetyltrimethyl ammonium bromide against *Bacillus cereus *vegetative cells and *Pseudomonas fluorescens*. The bacteria (single and consortium) were exposed to surfactants (single and combined) in the absence and presence of potential disinfection interfering substances. The antimicrobial effects of the surfactants were assessed based on the bacterial respiratory activity measured by oxygen uptake rate due to glucose oxidation. The tested surfactants were efficient against both bacteria (single and consortium) with minimum bactericidal concentrations ranging from 3 to 35 mg·L^−1^. The strongest effect was caused by humic acids that severely quenched antimicrobial action, increasing the minimum bactericidal concentration of the surfactants on *P. fluorescens *and the consortium. The inclusion of the other interfering substances resulted in mild interferences in the antibacterial activity. This study clearly demonstrates that humic acids should be considered as an antimicrobial interfering substance in the development of disinfection strategies.

## 1. Introduction 

In order to prevent and control microbial proliferation in industrial settings, cleaning and disinfection plans are applied on a regular basis [1, 2]. In food processing plants, the control of microbial contamination generally involves clean-in-place (CIP) procedures which consist of running alternated cycles of detergent and disinfectant solutions with water rinses in high turbulence regimes through the plant and pipeline circuits without dismantling or opening the equipment [2–5]. 

Biocides are currently used in industrial processes as the most significant countermeasure to control microbial growth and proliferation [6]. Industry moved progressively towards the use of surfactants that are less toxic and more biodegradable [7]. Surfactants are classified according to the ionic physiognomies of their hydrophilic group as anionic, cationic, nonionic, and zwitterionic [6, 8]. Quaternary ammonium compounds (QACs) are cationic surfactants that are commonly used because of their hard-surface cleaning, odor removal and antimicrobial properties [9]. Besides killing bacteria, the chemical nature of QACs can cause modifications on the properties of abiotic surfaces, decreasing their tension and therefore preventing attachment of microorganisms [7]. The antimicrobial mode of action of cationic surfactants is proposed by some authors as a sequence of events: attraction by the negatively charged cell surface; adsorption to the cell wall through the hydrophobic headgroup; reaction with the lipids and proteins that compose the cytoplasmic membrane; and cell penetration and interaction with intracellular constituents [10, 11]. Thus, QACs damage the outer layers of bacteria [9], thereby promoting the release of intracellular constituents [12].

Antimicrobial efficacy tests require planning of an adequate strategy and should include all the parameters found in real settings [13]. Aspects such as the proper contact time under known water hardness and conditions of high or low soil content should be considered [14]. For an effective cleaning and disinfection plan, the choice of the disinfectant must follow specific criteria such as compatibility with the surfaces to be disinfected, economic constraints, safety in the workplace, toxicological safety, and biological degradability [15]. It should, most of all, target the type of bacteria and the type of soiling [16]. In fact, disinfectants can be seriously affected by the presence of organic matter [17]. 

Interfering substances have been studied in the last years and included in cleaning and disinfection plans regulated by the authorities such as the European Standard EN-1276 [18]. There are already some reports on the effects of interfering substances in disinfection. However, most of these studies only address the effects of bovine serum albumin (BSA) and water hardness [9, 14, 15, 19–21]. Aal et al. [15] evaluated the bactericidal activity of disinfectants referred in the German Veterinary Society guidelines as references for testing disinfectants used in dairy and food industries. In order to simulate the conditions found in practice, they used low fat milk as an organic load and reported the significance in choosing an appropriate disinfectant since the inclusion of a challenging substance (organic material) is important to access the proper bactericidal activity. Bessems [14] demonstrated that a QAC tested on three microorganisms (*Pseudomonas aeruginosa*, *Staphylococcus aureus,* and *Candida albicans*) had a similar killing rate in the absence of interfering substances and after the inclusion of 17 dH water hardness, a strong reduction of the killing activity was found for the Gram-negative bacteria. However, the same behavior was not verified for the other two microorganisms. Jonõ et al. [19] assessed the effect of dried yeast and human serum on the activity of benzalkonium chloride and concluded that the bactericidal activity of the QAC was inhibited by solutions of both interfering substances. The inhibition by yeast extract was more pronounced than the inhibition by human serum.

This work provides information on the influence of potential interfering substances (bovine serum albumin—BSA, alginate—ALG, yeast extract—YE, and humic acids—HA) on the antimicrobial activity of two QACs (benzalkonium chloride and cetyltrimethyl ammonium bromide) against *Bacillus cereus* and *Pseudomonas fluorescens,* as they are two major contaminants in the food industry, particularly the dairy industry, and are a known cause of produce spoilage and foodborne illnesses [2, 22–26]. Some of the interfering substances used throughout the experiments are proposed in the European Standard EN-1276 [18] as potential interfering agents in disinfection while the others are extracellular polymeric substances (EPS) from the biofilm matrix that have an important role in antimicrobial resistance [27].

## 2. Materials and Methods

### 2.1. Microorganisms and Culture Conditions

The bacteria used in this work were* Pseudomonas fluorescens* ATCC 13525^T^ and a *Bacillus cereus* strain, isolated from a disinfectant solution and identified by 16S rRNA gene sequencing [28].

Bacterial strains were grown at a temperature of 30 ± 2°C and pH 7, with glucose as the main carbon source. Culture medium consisted of 5 g·L^−1^ glucose, 2.5 g·L^−1^ peptone, and 1.25 g·L^−1^ yeast extract in phosphate buffer (PB) (pH 7, 0.025 M) [29]. A bacterial suspension was prepared by inoculation of a single colony grown on solid medium into a 1 L flask containing 250 mL of sterile nutrient medium. This bacterial suspension was incubated overnight at the given temperature with agitation (120 rpm). 

### 2.2. QACs and Interfering Agents

The QACs used throughout the experiments were benzalkonium chloride (BAC) and cetyltrimethyl ammonium bromide (CTAB) (Sigma, Portugal). Preliminary studies with a concentration range between 0 and 5000 mg·L^−1^ were initially made. In order to ascertain the behaviour of bacteria to the QAC, the selected concentrations for further studies were 3, 5, 10, 20, and 35 mg·L^−1^. The QACs were used individually and in combination (both chemicals were combined in equal volumes and concentrations). 

The interfering substances used throughout the experiments were alginic acid sodium salt—ALG (Sigma, Portugal), bovine serum albumin—BSA (Sigma, Portugal), humic acids—HA (Acros organics, Fisher Chemical, Portugal), and yeast extract—YE (Merck, Portugal).

### 2.3. Disinfection Procedure

After the growth period, the suspensions were centrifuged (3999 *g*, 5 minutes), washed two times, and resuspended in PB to a final cell density of approximately 1 × 10^9^ cells·mL^−1^. In the case of the consortium, both bacterial suspensions were washed two times resuspended in PB to a final cell density of approximately 1 × 10^9^ cells·mL^−1^, and combined in equal volumes to obtain the same cell concentrations of the single species tests. Afterwards, all bacterial suspensions were exposed to several concentrations of QAC for a period of 30 minutes [30]. The effects of the chemicals were evaluated by the assessment of the oxygen uptake rate due to glucose oxidation, according to Simões et al. [30]. 

To investigate the influence of interfering substances on the antimicrobial efficacy, the same procedure was followed with the addition of 300 mg·L^−1^ of BSA, ALG, YE, or HA to the bacterial suspension, simulating low concentrations of interfering substances according to the European Standard EN-1276 [18]. Three independent experiments, each with duplicate samples, were performed for each condition tested.

### 2.4. QACs Neutralization

A neutralization process was performed after the disinfection procedure. The methodology was performed according to Johnston et al. [31] for a period of 10 minutes. BAC and CTAB were chemically neutralized by a sterile solution of (w/v) 0.1% peptone, 0.5% Tween 80, 0.1% sodium thiosulphate, and 0.07% lecithin dissolved in PB. All the chemicals were obtained from Sigma (Portugal). Control experiments were performed to ascertain the effects of the 10-minute exposure to the neutralization solution, and no effects were detected on the respiratory activity of *B. cereus* and *P. fluorescens* (data not shown). After the neutralization step, the bacterial suspensions were centrifuged (3999 *g*, 5 min) and resuspended in the same volume of PB.

### 2.5. Respiratory Activity Assessment

The respiratory activity was ascertained by measuring oxygen uptake rates in a biological oxygen monitor (Yellow Springs Instruments 5300A). Simões et al. [30] demonstrated that this procedure is more adequate and rapid than the assessment of colony forming units to characterize the antimicrobial activity of biocides against heterotrophic aerobic bacteria [21]. Samples were placed in the temperature-controlled vessel of the biological oxygen monitor (*T* = 25 ± 1°C) each containing a dissolved oxygen probe connected to a dissolved oxygen meter. Before measuring, the samples were aerated for 10 minutes to ensure oxygen saturation ([O_2_] = 8.6 mg·L^−1^). The vessel was closed, and the decrease of oxygen concentration was monitored over time. The initial linear decrease corresponds to the endogenous respiration rate. To determine the oxygen uptake due to substrate oxidation, 12.5 *μ*L of a 5 g·L^−1^ glucose solution was added to each vessel. The slope of the initial linear decrease in dissolved oxygen, after glucose injection, corresponds to the total respiration rate. The difference between these two rates is the oxygen uptake rate due to glucose oxidation [9].

The inactivation was calculated using metabolic activities according to the following equation:

(1) $$\begin{array}{c} \%\;\text{Inactivation}=\frac{\left(m_{c}-m_{t}\right)}{m_{c}}\times 100, \end{array}$$

where *m*_*c*_ is the metabolic activity of the control experiments (without antimicrobial exposure) and *m*_*t*_ is the metabolic activity of the bacterial solutions exposed to the antimicrobial. If % inactivation >0 there was inactivation of the microorganisms whereas if % inactivation <0 there was metabolic potentiation. 

The MBC for each situation was determined as the lowest concentration of QAC or QAC combination where no respiratory activity was detected [31].

### 2.6. Statistical Analysis

For each parameter tested the average and the standard deviation were calculated. The statistical significance of the results was evaluated using the Wilcoxon test (confidence level ≥ 95%) to investigate whether the differences between the resulting experimental values could be considered significant.

## 3. Results 

The antibacterial activity of BAC, CTAB, and their combination was investigated in the absence and in the presence of four selected interfering substances.

**Figure 1:**
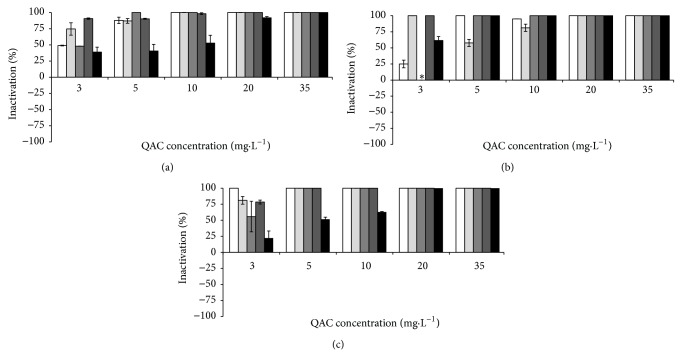
Inactivation of *B. cereus* by BAC (a), CTAB (b), and QAC combination (c), where solid white box is the control (no interfering substances), light grey box corresponds to BSA, grey box, is ALG dark grey box YE, and black box HA. ∗ means no inactivation. Mean values ± standard deviation for at least three replicates are illustrated.

**Figure 2:**
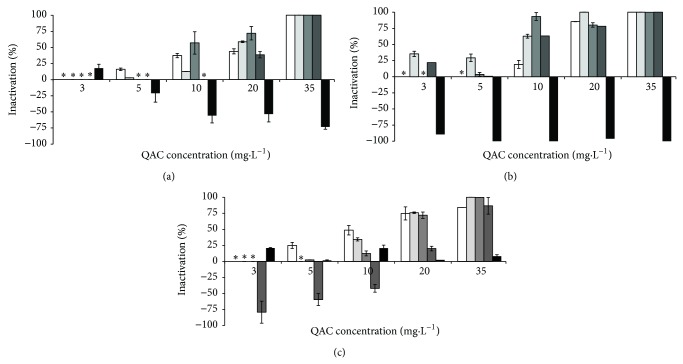
Inactivation of *P. fluorescens* by BAC (a), CTAB (b), and QAC combination (c), where solid white box is the control (no interfering substances), light grey box corresponds to BSA, grey box is ALG, dark grey box is YE, and black box is HA. ∗ means no inactivation. Values below zero are indication that the metabolic activity increased in comparison with the control experiment. Mean values ± standard deviation for at least three replicates are illustrated.

**Figure 3:**
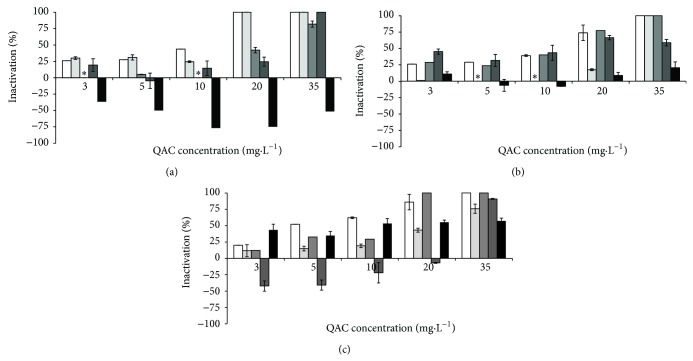
Inactivation of the consortiumby BAC (a), CTAB (b), and QAC combination (c), where solid white box is the control (no interfering substances), light grey box corresponds to BSA, grey box is ALG, dark grey box is YE, and black box is HA. ∗ means no inactivation. Values below zero are indication that the metabolic activity increased in comparison with the control experiment. Mean values ± standard deviation for at least three replicates are illustrated.

In the absence of interfering substances BAC caused the inactivation of *B. cereus* at 10 mg·L^−1^, *P. fluorescens* at 35 mg·L^−1^, and the consortium at 20 mg·L^−1^. CTAB at 20 mg·L^−1^ completely inactivated *B. cereus* and at 35 mg·L^−1^ inactivated the total population of *P. fluorescens* and the consortium. The combination of both QACs was synergistic in the inactivation of *B. cereus* (total inactivation with 3 mg·L^−1^) and indifferent for *P. fluorescens* (35 mg·L^−1^) and the bacterial consortium (35 mg·L^−1^). The inclusion of the selected interfering substances influenced the antimicrobial activity of the QACs to some extent (Figures 1–3). The inactivation of* B. cereus* (Figure 1) was not affected by the presence of any interfering substances (*P* > 0.05), except with HA. This interfering substance decreased the antimicrobial efficacy of BAC and the combination of QACs. The antimicrobial action of the QACs against *P. fluorescens* (Figure 2) was not significantly influenced by the presence of most potential interfering substances (*P* > 0.05), except for HA where interference was observed (*P* < 0.05). The antimicrobial activity of the QACs against the bacterial consortium (Figure 3) was affected by the presence of interfering substances. ALG and HA reduced significantly the activity of BAC (*P* < 0.05). HA reduced significantly the activity of CTAB at higher concentrations (*P* < 0.05). BSA and YE resulted in a significant reduction of the activity of the combination of QACs (*P* < 0.05).

Linear correlations were determined to assess the relationship between QAC concentrations and the inactivation data. The effect of increasing QAC concentration on bacterial inactivation shows that there are strong linear correlations (*R* > 0.850) for the control assays, with the exception of *B. cereus* (this bacterium was inactivated with low QAC concentrations). When interfering substances were added, the correlations decreased. The most extreme cases are the treatments with CTAB to *P. fluorescens* with ALG as an interfering substance (*R* = 0.771) and the bacterial consortium in the presence of YE (*R* = 0.738). Likewise, this decrease of linear correlation factors was found for *P. fluorescens* and for the consortium exposed to HA where the lowest correlation factor was 0.153, which was obtained for *P. fluorescens* treated with CTAB.

The results also demonstrate the occurrence of metabolic potentiation (inactivation below 0%). This phenomenon only happened when the QACs were used on *P. fluorescens* and the bacterial consortium in the presence of YE and HA. The most significant cases of oxygen uptake rate increase were verified for *P. fluorescens* exposed to BAC (5 to 35 mg·L^−1^) and CTAB (3 to 35 mg·L^−1^) in the presence of HA and combination of QACs (3 to 10 mg·L^−1^) in the presence of YE. A similar metabolic behaviour was found for the bacterial consortium exposed to BAC (3 to 35 mg·L^−1^) and CTAB (5 and 10 mg·L^−1^) for HA and QAC combination (3 to 20 mg·L^−1^) with YE.

The MBC values for the different conditions tested (single and combined QACs, in the absence and presence of potential disinfection interfering substances) are shown in Table 1. The presence of BSA increased the MBC of the combination of QACs for *B. cereus* (3 to 5 mg·L^−1^) and the consortium. ALG increased the MBC of BAC for the consortium (20 to over 35 mg·L^−1^) and QACs combination (3 to 5 mg·L^−1^) for *B. cereus*. YE increased the MBC of BAC for *B. cereus* (10 to 20 mg·L^−1^) and QAC combination (3 to 5 mg·L^−1^). *P. fluorescens* MBC increased with the inclusion of YE with the combination of QACs. The MBC values for the consortium of cells increased in the presence of YE (BAC—20 to 35 mg·L^−1^, CTAB—35 to over 35 mg·L^−1^, and QAC combination—35 to over 35 mg·L^−1^). HA increased the MBC for all the scenarios, except of CTAB when applied to *B. cereus* (in this situation the MBC was reduced). The MBC was reduced in other situations such as, for *B. cereus*, in the presence of ALG when using BAC and CTAB (10 to 5 mg·L^−1^ and 20 to 5 mg·L^−1^, resp.) and in the presence of YE when using CTAB (20 to 3 mg·L^−1^). *P. fluorescens* inactivation by CTAB was reduced by BSA (35 to 20 mg·L^−1^). ALG also reduced the antimicrobial activity of the combination of QACs against the bacterial consortium (35 to 20 mg·L^−1^).

## 4. Discussion 

In disinfection practices, the environmental characteristics can influence the antimicrobial activity of biocides [32]. It is assumed that the organic material can potentially interfere with the antimicrobial agents by chemical and/or ionic interactions [15, 33]. Therefore, it is necessary to know the role of each potential interfering substance in the antimicrobial activity in order to develop effective disinfection strategies. The interfering substances tested are commonly found as residuals in the food industry (from food products and from microbial contaminants, biofilms) [18, 27]. 

In this study, higher inactivation rates were verified for *B. cereus* in comparison to *P. fluorescens* at the same QAC concentration. The inactivation profiles of the cell consortium are similar to *P. fluorescens*. In fact, when *B. cereus* and *P. fluorescens* are combined in a 1 : 1 bacterial suspension, it is expected that the first is more affected than the second. *B. cereus* is more susceptible due to the fact that it is a Gram-positive bacterium that lacks an outer membrane, which typically provides increased protection to Gram-negative bacteria. This fact is corroborated by previous reports which stated that Gram-positive bacteria are more susceptible to cationic surfactants than Gram-negative bacteria [34, 35].

BSA was already studied as an interfering substance in disinfection practices [9, 14, 19–21, 36]. The negative effect of BSA on the action of biocides against *P. fluorescens* was demonstrated by Simões et al. [9, 21]. *P. fluorescens* treatment with CTAB with the addition of 3 g·L^−1^ of BSA resulted in a 10-fold increase on the MBC of this QAC [9, 21]. In the present study, low BSA concentrations decreased the antimicrobial activity of the QACs. The efficacy of the combination of QACs against *B. cereus* and the cell consortium was also reduced. This effect of BSA as an antimicrobial quencher is apparently due to the strong ability of QACs to react with proteins [21]. Proteins can precipitate in the form of their anions, in this way, the negative-charged protein ions will cling to the positively charged molecules of the cationic compounds [37]. CTAB is a biocide that targets the membrane and has a strong affinity for proteins [21]. BAC is composed of a positively charged hydrophobic headgroup which clings to opposite charged surfaces [8, 37]. Jonõ et al. [19] studied the effect of the alkyl chain of BAC binding to BSA and dried yeast. Their conclusions were that BAC is often inactivated by organic matter, either by adsorption to the bacterial surface or by adsorption to the organic matter in general. These authors also suggested that the reduction in the activity of BAC was probably related to more than one physical property of the compounds like the chain length (longer chains result in more adsorption to the bacterial surface). 

ALG is a common constituent of the extracellular polymeric substances of the biofilm matrix [38–40]. A function frequently attributed to EPS is their general protective effect on biofilm microorganisms against adverse conditions. The EPS matrix delays or prevents antimicrobials from reaching target microorganisms within the biofilm by diffusion limitation and/or chemical interaction with the extracellular proteins and polysaccharides [32, 41]. In this study, ALG either potentiated or hindered the antimicrobial activity of the selected QACs. The presence of this interfering substance was not obvious on the inactivation of *P. fluorescens*. On the other hand, the inactivation of *B. cereus* by BAC and CTAB and the consortium by the combination of QACs was easier in the presence of this interfering substance. The bacterial consortium treatments with BAC and *B. cereus* with the combination of QACs were hampered by the presence of ALG. Davies et al. found that the production of ALG was triggered by membrane perturbation induced by ethanol stress, nitrogen limitation, attachment to surfaces, or even high oxygen tension [42, 43]. This substance is suggested as one of the main biofilm resistance vectors either by reacting with the antimicrobials or by hindering antimicrobials diffusion to the cells [44]. The antimicrobial interference caused by ALG is apparently due to electrostatic interactions between the anionic ALG and the cationic-selected QACs [45]. 

The presence of YE as interfering substance resulted in three different outcomes on the antimicrobial activity of the QACs: (1) no effect/indifference, (2) the respiratory activity reduced, and (3) the respiratory activity potentiated. This interfering substance worked mainly as a hinderer of the antimicrobial activity by increasing the MBC of *B. cereus* in all cases except for CTAB, of *P. fluorescens* with the combination of QACs, and of the consortium of cells with CTAB and the combination of QACs. These results are in accordance with the available studies. YE is listed in the European Standard EN-1276 as an interfering substance native to the brewery industry [18]. The constituents of YE are very similar to the components of the bacterial cells, thus, it is expected that the antimicrobial agents that target the bacterial cells are also drawn to YE. In a similar study by Jonõ et al. [19] it was shown that the presence of dried yeast decreased the biocidal effectiveness of BAC. 

Humic substances are found ubiquitously in the environment and can be found in the biofilm matrix [2, 46]. HA reduced the antimicrobial activity of the QACs in most of the cases, although in some cases it promoted the respiratory activity (potentiation). The presence of these compounds had the strongest effect compared to the remaining interfering substances. Like ALG, HA are known to be a part of the EPS composition [47]. Atay et al. [8] studied the sorption mechanisms of anionic and cationic surfactants to natural soils concluding that the dominant sorption mechanism of surfactants to clay is cation exchange. Ishiguro et al. [48] reported that cationic surfactants bind strongly to humic substances. Koopal et al. [49] also verified the formation of complexes HA-cationic surfactant. These observations are consistent with the present results. 

Respiratory activity potentiation was verified with the addition of HA to *P. fluorescens *and YE to the bacterial consortium. It is known that HA participates in cellular metabolism processes such as growth, respiration, photosynthesis, and nitrogen fixation [50]. On the other hand, HA were proposed to replace synthetic surfactants such as SDS, Tween 80, and Triton X-100 in industrial applications such as textile dying or washing [51]. It is therefore possible that the inclusion of humic substances in a solution of QACs may interfere with the chemical characteristics of the solution. The resultant mixture, with an apparent reduced antimicrobial efficacy, seems to potentiate the respiratory activity of the bacteria, particularly of *P. fluorescens*. As QACs are membrane active agents, their use at sublethal concentrations could improve membrane permeability and consequently the nutrient influx, without compromising the bacterial viability. Also, there is the hypothesis that the potentially interfering agents could be used as nutrients. In fact, it was found that the growth rates of anaerobic and aerobic microorganisms increased when humic substances were added, which stimulated enzyme activity [52, 53]. In a similar way, YE is a nitrogen source widely used as a component of growth media [54]. HA are likely to be used for growth in the same way as YE; these might be broken down to smaller molecules that can be used by cells as a carbon [55] or nitrogen sources [51].

The antimicrobial activity of the tested QACs was enhanced in some cases, where the interfering substances were present. This is an unexpected result due to the recognized potential of ALG, BSA, HA, and YE to interfere with disinfection. This effect is probably due to the low concentration of interfering substances tested that caused both respiratory activity reduction and potentiation. Cases of antimicrobial enhancement are widely known. Ethylenediamine tetraacetate (EDTA) was reported as early as 1965 to increase the biocidal effects of BAC and chlorhexidine diacetate on *Pseudomonas aeruginosa* [56]. Sagoo et al. [57] reported that chitosan (a polysaccharide) potentiated the antimicrobial action of sodium benzoate on spoilage yeasts. In dairy plants, disinfection is potentiated by pre-washes with alkali or enzyme-based cleaning agents [58]. The antimicrobial potentiation of the QACs occurred in some cases. Most of these cases were observed for *B. cereus* (four occurrences), one was observed for *P. fluorescens,* and another one was observed for the consortium of cells. The MBC was improved by more than 50% in the cases of *B. cereus* and less than 30% for *P. fluorescens* and the consortium of cells. To our knowledge there are no reported cases of antimicrobial agents potentiation by BSA, YE, or ALG. Concerning the effects of HA, these molecules are reported to have detergent properties [51]. Although the exact chemical structure of HA has not yet been determined, HA could be chemically similar to the tested QACs, presenting a positive hydrophilic head and a hydrophobic tail. With this structure HA could act as detergents in conditions such as those observed in the treatment of *B. cereus* with CTAB [51].

The present work shows that increasing QACs concentrations lead to an increase in antimicrobial effectiveness. This is valid mainly when the QACs were applied in the absence of interfering substances. This means that disinfection was concentration dependent, as found for most of the antimicrobial chemicals [59]. However, the linear dependency of inactivation versus concentration is not verified for most of the tests where interfering substances were added. This result evidences that the mathematical modelling of disinfection strategies requires a case-to-case analysis when interfering substances are present.

## 5. Conclusions

The overall results demonstrate that a disinfection process in the presence of the selected interfering substances can reduce the effectiveness of BAC, CTAB, and their combination. The bacteria were inactivated equally by all QACs, although in the absence of interfering substances CTAB was the most efficient solution. *P. fluorescens* was the bacterium with the highest resistance to inactivation, followed by the bacterial consortium. The tested interfering substances, referred in the European Standard 1276 (BSA and YE), and known EPS constituents related with biofilm resistance (ALG) resulted in mild interferences on the activity of the QACs. HA were the interfering substance that resulted in the most severe effect by reducing the activity of QACs, causing, in some circumstances, significant respiratory activity potentiation. This interfering substance should therefore be considered when developing disinfection protocols.

## Figures and Tables

**Table 1:** Minimum bactericidal concentration for *P. fluorescens*, *B. cereus,* and the consortium with and without interfering substances.

MBC (mg·L^−1^)				
		BAC	CTAB	QAC combination
Control	*B. cereus *	10	20	3
	*P. fluorescens *	35	35	35
	Consortium	20	35	35

BSA	*B. cereus *	10	20	5
	*P. fluorescens *	35	20	35
	Consortium	20	35	>35

ALG	*B. cereus *	5	5	5
	*P. fluorescens *	35	35	35
	Consortium	>35	35	20

YE	*B. cereus *	20	3	5
	*P. fluorescens *	35	35	>35
	Consortium	35	>35	>35

HA	*B. cereus *	35	5	20
	*P. fluorescens *	>35	>35	>35
	Consortium	>35	>35	>35
